# Allegheny County Medical Society

**Published:** 1895-09

**Authors:** 


### *SOCIETY REPORTS.*

### ALLEGHENY COUNTY MEDICAL SOCIETY.

PITTSBURG, PA., May 28, 1895.

The general subject for discussion was "The Relation of the Medical Profession to the Courts, with Special Reference to Expert Testimony in Cases of Insanity."

The first paper was read by DR. THEODORE DILLER, entitled "What Shall be Done with the Homicidal Crank?" (See abstract in this issue.)

DR. C. C. HERSMAN presented a paper on "Insanity and the Courts." He said that the legal profession, particularly the judiciary, were accustomed to look only to the *lex scripta;* to inquire what the law really is, rather than to investigate its justice, its reasons, its principles.

As to the true tests of legal responsibility in the insane, law is (in the opinion of the medical profession at least) very short of what the science of medicine demands. The unprejudiced mind should investigate this subject in the light which science has brought to its solution. Our lawmaking power should examine our statutes to see if they are founded upon sound principles.

The medical profession, yea, all professions, the public even, know that a knowledge of right and wrong, and of the penalties of the law, are not the proper tests in such cases. The men best qualified to judge tell us that the insane not only know right from wrong and often fully understand the nature and penalties of the law, but as a rule they can discriminate between right and wrong in their wrongdoings committed under the force of insane delusions which they cannot resist. These truths must be considered in determining criminal responsibility.

The legal profession, especially the thinking members, must acknowledge this. They must also acknowledge that our law, as interpreted by our courts, in many cases is misleading; and that a careful revision by our legislators would be a speedy relief.

The writer then related the case of George Ducovic, tried and convicted of murder in Pittsburg, April, 1894. He had examined the prisoner after the conviction and was sure he was insane. He had had delusions of suspicion and persecution, and killed his victim in order (as he thought) to save his own life and to rid the community of a dangerous character. His was a typical case, in that these persons reason correctly from false premises; their statements are coherent and rarely confused. With a fixed delusion such a one may go on for years making a living and possibly accumulating something; but, as a rule, he is lacking in effort in one direction and apt to be meddlesome. He is thought to be harmless. A jury would likely see only eccentricity, "just a little off," and the populace go on unconscious of danger until something desperate is done to avenge an imaginary wrong. He thinks he shall be a public benefactor if he rids the world of this culpable individual. Encouraged by the hope of notoriety, he expects to gain the highest pinnacle of publicity and be hailed as a great deliverer. Does he know that he is wrong? Could he resist the insane desire to act? Unquestionably no. These cases do not attribute their annoyance to unnatural or unseen things, or impossible means, but to the malevolence of real persons who plot against them, have evil designs on them, who poison their food, etc.

That the prisioner knew he was killing a man and that he knew the penalty, does not exclude insanity, for I have had under my care the worst kind of insane who knew the penalty of murder, yet they were mad. In any other case than murder, an irrational act is taken as ground at least for suspicion that the mind of the perpetrator is disordered, but in murder no account is taken of the unreasonable act.

The medical profession may be said to agree substantially as a body, that in the homicides by the insane a knowledge of the character of the act committed, and of its being a violation of law, is not a safe and reliable test of responsibility. We have investigated this question with courage, without prejudice, and in the light which science has brought to the solution. There is too much belief in the common sense of a jury, to be the best judge of insanity. One great fault is partly due to the law requiring definitions from medical witnesses.

We can no more define insanity than we can by definition give an expression of a rainbow or landscape. Except in a few cases insanity does not develop suddenly. "It does not begin and end with a criminal act as its sole manifestation." The act when committed is but one of several symptoms of his disease. In the case of Ducovic, in coming to a conclusion in our report to the governor, we considered the whole group of symptoms to amount to insanity. In his own solitude, the physician will have less difficulty to demonstrate the fact that the accused is insane, than when he is subjected to the irregularities of cross-examination by lawyers.

For the trial of such cases the author recommended the selection of a commission of competent medical men to sit with the judge in an advisory capacity. Such a commission would be independent and free from bias. Such a procedure is used in certain English courts, and has been used in one case (Schneider, Washington, D. C.) in this country.

DR. SAMUEL AYERS read a short paper suggesting "How a Higher Standard of Medical Expert Testimony may be Obtained." He suggested these methods:

In the first place, the selection by the court, or by some judicial authority, in every case requiring expert medical testimony, of a commission of three competent physicians who shall be allowed every opportunity to examine the case or the evidence, and who shall, after full consultation, render their opinion to the court, would, in my judgment, be a means of elevating the standard of such testimony.

Secondly, only such physicians as are well qualified from experience and knowledge in their special branch should be selected by lawyers or others to give expert testimony.

In the third place, it is a duty physicians owe their own consciences, the community, and the profession itself, that they do not consent to testify until they have thoroughly examined into the merits of a legal case, and then testify only in conformity with their convictions.

DISCUSSION.

JUDGE EWING was present by invitation and said: When I came to this meeting I supposed that I would have an opportunity of hearing your opinions of the legal profession and of courts, rather than to express an opinion of the medical profession or of their beliefs. The doctors often learn in court what the lawyers think of them, and sometimes what the judges think of medical opinions; and I expected to hear an expression from the doctors, this evening, of their estimate of lawyers and judges, and have not been disappointed.

Now I did not come prepared to discuss this subject of insanity, with its bearing on legal responsibility. I came to learn something. The question is a very broad one. I have been greatly interested in the papers read, but I cannot, however, agree with many of the views advanced, and especially with regard to giving the disposal of criminal, especially of homicide, cases over to experts alone, or to take their testimony as conclusive. In my opinion, this would be a very dangerous departure—the specialist and expert is so liable to become one-sided, whether he be a medical expert, a patent expert, or whatever it may be. If I had a friend or relative whom I suspected was of somewhat unsound mind, or had some mental trouble, and I wanted to cure him, I would probably go to a specialist, an expert on that line of disease; but if I wished to be satisfied as to whether or not he was mentally sound, that is, sound enough to be morally and legally responsible for his acts, I would be disposed to go to the good old-fashioned, "all-around" country doctor, who knows a little of everything.

I think in may cases of alleged insanity the expert goes on the stand with a wrong impression of what he is there for. He is liable to think he is there to decide questions of law, and at times the expert witness would be of more assistance to judge and jury if that idea could be eliminated. This erroneous idea is also prominent in medical works. Two or three years ago, in a case tried before me, the jury brought in a verdict of murder of the first degree. The verdict was against the testimony of the experts. I consulted a number of medical works on the subject of the particular phase of insanity from which it was alleged the defendant was suffering, to get more light on the subject, in order to satisfy my conscience and my judgment as to what I ought to do. From some of these books I received very little assistance. I found the same difficulty—in nearly all the books the writers seemed determined to discuss and decide the law, rather than to give the facts or give the nature and effect of the disease in its different phases. I doubt if the expert in insanity is ever the most competent man to judge and decide what the law should be. In only two of about ten books which I consulted did I find anything of assistance to me. I remember one of them especially, in which the author deemed it his province to lay down the law, about which he knew nothing.

JUDGE SLAGLE said: "The law books, as well as the medical books, say that delusion is insanity. It is not merely evidence of insanity. It is insanity. The questions for the lawyers, the judges, and the juries to decide is whether or not that insanity controls the man's will to such an extent that he cannot resist it. If the insanity reaches that point, then responsibility ceases. If the preversion of mind governs the man's entire actions he is totally insane. But he may be sane on some subjects and perfectly insane on others, and if the act that is done is in the line of insane delusion, he is not responsible morally or civally. When does responsibility cease? That is the question. As Judge Ewing states, the doctors are to determine whether the man has an insane delusion; the facts and law of that insane delusion belong to the judge and the jury. We have the expert opinion to guide the jury, we have the judge to instruct them and to explain and apply the law, and I think that justice can be done as effectually in this manner as by a commission of experts. And this brings me to the consideration of a point in Dr. Diller's paper on the subject of cranks. The question in such case is, whether the man's peculiar ideas or sentiments constitute an insane delusion. If it is not an insane delusion but merely a perversion of judgment, the man may be wicked, but he is not insane. Such cranks as Guiteau or Prendergast and the like do not come under any rule that exempts them from punishment for their crimes. Ask these men the reasons for their crime. They will tell you they are working for the good of society, that they do not care for themselves. We might say this is a crazy delusion, but these were wicked men rather than insane. Booth possibly thought he was doing his country service in killing Lincoln, but his mistaken judgment must not excuse his crime. I was very much interested in the articles read to-night. I think that the free discussion of such articles will be productive of much good. And I would recommend now, as I have done before, that lawyers and physicians should meet together and discuss such subjects. It would do both good.

FRANK I. GOSSER, Esq.: I have noticed a feeling among medical gentlemen on and off the stand, a feeling of unrest and dissatisfaction arising from the consciousness that the members of their profession as expert witnesses are not accorded the consideration which they believe is their right, and I have heard considerable complaint on this score. The fact that their position, and their importance, and their learning have been minimized by the court, and that sufficient weight is not attached to their opinion as scientific men, are sources of dissatisfaction and annoyance. But I must coincide with the view of Judge Slagle. I do not think there has been anything advanced by the medical gentlemen to-night that would improve the administration of the criminal justice, although the articles were of great interest. The question of determining the insantity of criminals by a commission is becoming to be a much mooted question. A commission of experts as an adjunct to the court in an advisory capacity might be an advantage, but I doubt the propriety of making the commission a tribunal. A commission of this kind might have been an advantage in the case of Frank Gerade, to which Judge Ewing referred. The accused was twice convicted of murder in the first degree, and the judge not only was compelled to make extensive research among medical authorities, but in order to gain more insight into the case, he determined to observe the conduct and actions of the prisoner in every way possible, to be better able to come to a just determination as to insanity of the accused, or whether he was feigning; and thus, by the vigilance and conscientiousness of the judge, the life of the accused was spared, and the administration of justice was exempted from the odium of having sanctioned the execution of a crazy man. As I said, in a case of this kind the assistance of an expert medical commission might be of advantage, but only as an adjunct, an accessory.

DR. ADOLPH KŒNIG: With all due respect to the opinions of the legal gentlemen concerning the relative merits of jury trials or commissions in these cases, I feel it my duty to express an opinion contrary to theirs. I cannot understand how a jury can be expected to give a just and intelligent opinion or verdict upon grave questions of the nature of which they know absolutely nothing. So far as I have followed jury trials, mainly through the columns of the daily newspapers, it has often seemed to me that very great injustice has been done. As far as I have been able to judge, I believe the juries are swayed to one side or the other by an eloquent attorney more readily than by the expression of scientific facts by scientific men. You may take, for instance, the case spoken of by Judge Ewing this evening, wherein all the facts were set before the jury and the man was proved to be insane, yet they found him guilty of murder. The fact that all the evidence of science was unable to set aside that verdict seems to me to be sufficient evidence to warrant the justice of leaving the disposition of these cases to a scientific, unbiased commission. Trial by commission seems to me to be the only way to arrive at a just decision.

I think even the legal profession will agree that all, or nearly all, the grand discoveries in the domain of medicine and surgery have been made by specialists, and not by the good old-fashioned country doctor. A man with a good brain, educated along one line of science, will surely accomplish more in that special line than a general practitioner. It seems to me if I were guilty of malpractice, and on trial, I would stand a much better chance of escaping punishment, if such were my motive, were I tried before a jury of ignorant men, with an eloquent lawyer to defend me, than I would have before an intelligent commission of scientific men.

DR. J. M. MURDOCH: Having had George Ducovic, the insane man of whom Dr. Hersman spoke, under my immediate charge at the Dixmont asylum, it may be in order for me to say that his conduct during the past months has fully justified the opinion of the experts who pronounced him insane, and he is more insane at the present time than he was at the time the experts made their decision. He is very eccentric in his manner of conducting himself and of wearing his clothes. He expresses a decided desire to get away from the institution in order to get an opportunity to shoot a man for his detention in the asylum. I agree with Dr. Diller's opinion that the best disposition of the criminal or homicidal crank is to place him under restraint in an insane asylum. I think there are two good and sufficient reasons for this. First, to protect society; second, that he may receive the benefit of the best scientific treatment, as a result of which there is a possibility of a restoration to his right mind and liberty to again return to his former mode of life.

DR. THEODORE DILLER: I wish to enter a protest against the stand taken by some of the legal profession to-night, and which is generally held by lawyers. They ask us to define exactly the line where responsibility ceases. A man has an insane delusion. That delusion is very marked. Does it certainly control his actions at the time he commits a crime? They say that responsibility ceases at the exact point where the delusion becomes supreme, and they ask us to state where the exact line is drawn. I do not think the expert should be expected to draw such a fine line. It is asking too much of him. They say that a man may have a delusion and yet be sane on some subjects and may commit a sane crime. I think this proposition is absurd. Who can tell whether the crime was committed in a sane or an insane moment. The benefit of the doubt ought always to be given to the insane man, or the man with the delusion or hallucination. If insane at all, then it should be assumed that any crime was a piece of insane conduct. I must say that I think the appointment by the court of an unbiased commission of experts to pass upon insanity cases would be of great value. This has been proposed in the State of Illinois.

